# Novel Prophylactic Vaccine Using a Prime-Boost Method and Hemagglutinating Virus of Japan-Envelope against Tuberculosis

**DOI:** 10.1155/2011/549281

**Published:** 2011-03-07

**Authors:** Masaji Okada, Yoko Kita, Toshihiro Nakajima, Noriko Kanamaru, Satomi Hashimoto, Tetsuji Nagasawa, Yasufumi Kaneda, Shigeto Yoshida, Yasuko Nishida, Hitoshi Nakatani, Kyoko Takao, Chie Kishigami, Shiho Nishimatsu, Yuki Sekine, Yoshikazu Inoue, David N. McMurray, Mitsunori Sakatani

**Affiliations:** ^1^Clinical Research Center, National Hospital Organization, Kinki-Chuo Chest Medical Center, 1180 Nagasone, Kitaku, Sakai, Osaka 591-8555, Japan; ^2^Ikeda Laboratory, GenomIdea Inc.,1-8-31 Midorigaoka, Ikeda, Osaka 530-0043, Japan; ^3^Division of Gene Therapy Science, Graduate School of Medicine, Osaka University, 2-2 Yamadaoka, Suita, Osaka 565-0871, Japan; ^4^Department of Medical Zoology, Jichi Medical School, 3311-1 Yakushiji, Minamikawachi-machi, Tochigi 329-0498, Japan; ^5^System Health Science Center, College of Medicine, Texas A&M University, College Station, TX 77843-1114, USA

## Abstract

*Objective*. *Mycobacterium tuberculosis* infection is a major global threat to human health. The only tuberculosis (TB) vaccine currently available is bacillus Calmette-Guérin (BCG), although it has no efficacy in adults. Therefore, the development of a novel vaccine against TB for adults is desired. *Method*. A novel TB vaccine expressing mycobacterial heat shock protein 65 (HSP65) and interleukin-12 (IL-12) delivered by the hemagglutinating virus of Japan- (HVJ)- envelope was evaluated against TB infection in mice. Bacterial load reductions and histopathological assessments were used to determine efficacy. *Results*. Vaccination by BCG prime with IgHSP65+murine IL-12/HVJ-envelope boost resulted in significant protective efficacy (>10, 000-fold versus BCG alone) against TB infection in the lungs of mice. In addition to bacterial loads, significant protective efficacy was demonstrated by histopathological analysis of the lungs. Furthermore, the vaccine increased the number of T cells secreting IFN-*γ*. *Conclusion*. This vaccine showed extremely significant protection against TB in a mouse model, consistent with results from a similar paper on cynomolgus monkeys. The results suggest that further development of the vaccine for eventual testing in clinical trials may be warranted.

## 1. Introduction

Tuberculosis (TB) is a major global threat to human health, with about 2 million people dying every year from *Mycobacterium tuberculosis* infection. The only TB vaccine currently available is an attenuated strain of *Mycobacterium bovis*, bacillus Calmette-Guerin (BCG), although its efficacy against adult TB disease is unclear. Furthermore, multidrug-resistant TB (MDR-TB) and extremely drug-resistant TB (XDR-TB) are becoming big problems worldwide. For these reasons, a prophylactic and therapeutic vaccine against TB is sought. TB vaccines are classified into 4 main groups: (1) DNA vaccines, (2) recombinant BCG vaccines, (3) subunit vaccines, and (4) attenuated vaccines.

It is well established that protective immunity to *M. tuberculosis* depends on both CD4^+^ and CD8^+^ T cells [1–6]. DNA vaccines induce cellular immune responses, including the Th-1-type cellular immune response, and they prevent infections in animal models [7, 8]. In fact, several human clinical trials have recently been initiated to test the efficacy of DNA vaccines against emerging and re-emerging infectious diseases including hepatitis B [9], malaria [10–12], and HIV infections [13]. DNA vaccines have also shown their potential as TB vaccines in mouse models [14–17]. However, in a guinea pig model, which is one of the most biologically relevant systems available for studying human pulmonary TB, DNA vaccines have not been proven more efficacious than BCG [18]. The efficacy of any experimental TB vaccine must be evaluated in human clinical trials, and a vaccine against TB is still anxiously awaited.

We have been developing a novel TB vaccine that is a DNA vaccine expressing mycobacterial heat shock protein 65 (HSP65) and interleukin-12 (IL-12), delivered by the hemagglutinating virus of Japan- (HVJ)- liposome or -envelope (HVJ-E) (HSP65 + IL-12/HVJ) [19–22]. The former vaccine was 100-fold more efficacious than BCG in a murine model on the basis of the elimination of *M. tuberculosis* [19]. In the present study, we demonstrated that the combination of BCG prime with HSP65 + IL-12/HVJ-E vaccine-boost was 10,000-fold more efficacious than BCG alone in a murine TB prophylactic model.

## 2. Materials and Methods

### 2.1. Bacteria

*M. tuberculosis* strains H37Rv and *M. bovis* BCG Tokyo, were kindly provided by Dr. I. Sugawara (JATA, Tokyo, Japan). *M. bovis* BCG Tokyo was maintained in synthetic Sauton's medium (Wako Chemicals, Osaka, Japan). For the mouse infection studies, a single colony of *M. tuberculosis* H37Rv was grown in Middlebrook 7H9 medium (DIFCO Laboratories, Detroit, MI; lot 137971 XA MD) supplemented with albumin-dextrose complex and grown at 37°C until approximately midlog phase. Aliquots were stored at −80°C and thawed 10 days before use. Each bacterium was grown to midlog phase in 7H9 medium.

### 2.2. Animals

Inbred and specific pathogen-free female BALB/c mice were purchased from Japan SLC (Shizuoka, Japan). Mice were maintained in isolator cages, manipulated in laminar flow hoods, and used between 8 and 10 weeks of age. All animal experiments were approved by the National Hospital Organization Kinki-chuo Chest Medical Center Animal Care and Use Committee. All vaccinations and experiments on isolated tissues were performed on anesthetized animals with sevoflurane. Infected animals were housed in individual microisolator cages in Biosafety Level (BL) 3 animal facility of the NHO Kinki-chuo Chest Medical Center.

### 2.3. Plasmid Construction

The *HSP65* gene was amplified from *M. tuberculosis* H37Rv genomic DNA, and cloned into pcDNA3.1 (+) (Invitrogen, San Diego, CA) to generate pcDNA-hsp65 (designated as HSP65 DNA) as described previously [19]. The *hsp65* gene was fused with mouse Ig*κ* secretion signal sequence, and pcDNA-Ighsp65 (designated as IgHSP65 DNA) was generated. For construction of the mouse IL-12 (mIL-12) *p40* and *p35* single-chain genes, *mIL12p35 *and* mIL12p40* genes were cloned from pcDNA-p40p35 [21], fused and cloned into pcDNA3.1 (+) to generate pcDNA-mIL12p40p35-F (designated as mIL-12 DNA).

### 2.4. HVJ-E Vaccination

**Figure 1:**
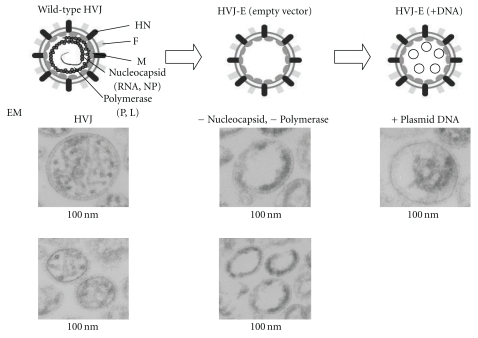
Hemagglutinating virus of Japan- (HVJ)- envelope vaccination: pcDNA3.1/HSP65DNA + IL-12DNA were incorporated into an HVJ-envelope empty vector (nonviral vector). Graphical representations of the HVJ-envelope empty vector in the presence or absence of DNA are shown. Electronic microscopy (EM) photographs of the HVJ-envelope empty vector are also shown.

HVJ-E was prepared as described previously (Figure 1) [19–23]. The HVJ-E complex was aliquoted and stored at −70°C until use. Groups of BALB/c mice were vaccinated 3 times at 3-week intervals with 100 *μ*L of HVJ-E solution containing 50 *μ*g of pcDNA-IgHSP65 and 50 *μ*g of mIL12 DNA. These DNA vaccines were injected into both anterior muscles in the tibia. Mice were vaccinated using 1 × 10^6^ colony-forming units (CFU) *M. bovis* BCG Tokyo by subcutaneous injection at 4 different sites (left-upper, right-upper, left-lower, and right-lower back). HVJ-E DNA vaccine containing pcDNA-IgHSP65 and -mIL12 DNA was designated as IgHSP65 + mIL-12/HVJ-E in this text.

### 2.5. Challenge Infection of Vaccinated Animals and Bacterial Load Determination

**Figure 2:**
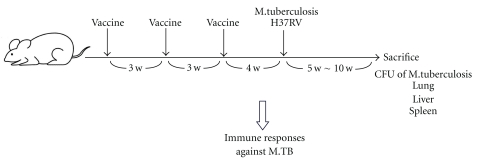
Groups of mice were vaccinated 3 times every 3 weeks using the prime-boost method and challenged intravenously with *M. tuberculosis* H37Rv as described in the Materials and Methods section. Five or 10 weeks after challenge with TB, protection was measured by enumerating bacterial loads (CFU) in the lungs, liver, and spleen of the vaccinated mice.

Mice were challenged by the intravenous route with 5 × 10^5^ CFU of *M. tuberculosis* H37Rv 4 weeks after the third vaccination as described previously (Figure 2) [19, 24]. 0.2 mL of saline containing 5 × 10^5^ CFU of H37Rv *Mycobacterium tuberculosis* were injected into tail vein of mice. At 5 and 10 weeks after *M. tuberculosis* H37Rv challenge, lungs, spleens, and livers were aseptically homogenized by using a homogenizer in saline, and serial dilutions of the organ homogenates were plated on 7H11 Middlebrook agar (Kyokuto. Tokyo, Japan). Plates were sealed and incubated at 37°C, and the number of colonies was counted 2 weeks later. Results were converted to log_10_ values. The log_10_ [mean ± standard deviation (S.D.)] values for CFU/organs/animals were calculated for each experimental group. Weight of lungs, liver, or spleen was measured by a balance (Sartorius Co. LP620S).

### 2.6. Histological Analysis

The lungs obtained from the mice were fixed with 10% buffered formalin and embedded in paraffin. Each block was sliced into 4-*μ*m-thick sections and stained using hematoxylin and eosin. Semiquantitative morphometric analysis of pathological slides was performed by a method modified over that of Dascher et al. (2003) using a micrometer-attached microscope (Microphot-FXA, Nikon, Japan) [19, 25, 26]. The longer axis and minor axis of each granuloma in the field (×40 magnification) were measured and then multiplied and summed. Three random fields from each tissue section of mice were evaluated. The average score of the fields was designated as the granuloma index (×10^−2^ mm^2^). This method for the evaluation of granuloma area was significantly correlated with the granuloma area determined by a hematoxylin and eosin section scanning method.

### 2.7. ELISPOT Assay

The spleens were removed aseptically from vaccinated mice 3 weeks after the third vaccination. Antigen-specific IFN-*γ*-producing cells were determined by enzyme-linked immunosorbent spot (ELISPOT) as described previously [19]. Briefly, ELISPOT plates (MultiScreen IP Filtration plate MAIPS45; Millipore, Bedford, MA) were coated with antimouse IFN-*γ* MAb R4-6A2 (BD Biosciences Pharmingen. San Diego, CA). Spleen cells from vaccinated mice were suspended at 1 × 10^7^ cells/mL (1 × 10^6^ cells/well). The cells were placed into 6 antibody-coated wells, and rHSP65 protein (10 *μ*g/mL) or PPD (10 *μ*g/mL) was added to each well. After 20 h of incubation at 37°C, cells were removed by washing the plates, and the site of cytokine secretions was detected using biotinylated antimouse IFN-*γ* MAb XMG1.2 (BD Biosciences Pharmingen) and streptavidin-alkaline phosphatase conjugate (BD Biosciences Pharmingen). The enzyme reaction was developed with BCIP-NBT substrate (Vector Laboratories Inc., Burlingame, CA). Spot-forming cells (SFCs) were enumerated using the KS ELISPOT system (Carl Zeiss, Hallbergmoos, Germany).

### 2.8. Statistical Analysis

Dunnett's tests (multiple comparisons) were used to compare log_10_ values of CFUs between groups following challenge and used to compare T-cell responses between groups in the ELISPOT assay. A *P*-value of <.05 was considered significant.

## 3. Results and Discussion

### 3.1. Results

#### 3.1.1. Prophylactic Efficacy Using Prime-Boost Method

The IgHSP65 + mIL-12/HVJ-E and BCG vaccines were administered using the prime-boost method as shown in Table 1. 

**Figure 3:**
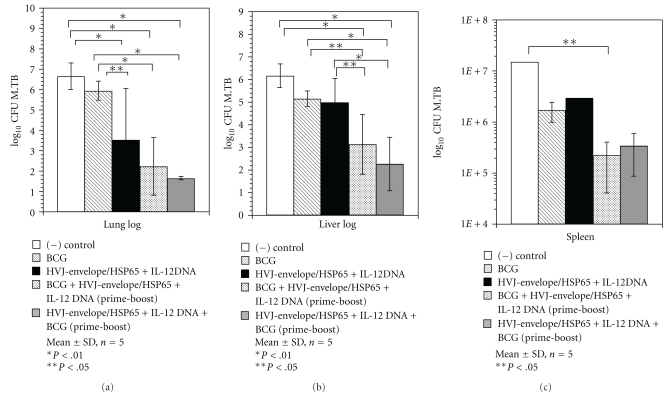
(a) Mouse protection studies using the prime-boost method. Groups of mice vaccinated with HVJ-envelope (HVJ-E) DNA and/or BCG were challenged by intravenous injection with *M. tuberculosis* H37Rv. Five weeks after challenge, protection was measured by enumerating the bacterial loads (CFU) in the lungs. Results are expressed as the mean log_10_ ± S.D. of CFU. The statistical significance of differences between individual groups in the CFU number was determined by Dunnett test (*n* = 5); *P < .01 and **P < .05; the statistical significance of differences (*P* < .01) of the G1 (naive) group compared to the G3 group (DNA/DNA/DNA), G4 group (BCG/DNA/DNA), or the G5 group (DNA/DNA/BCG). The statistical significance of differences (**P < .05) of the G2 group (BCG-alone group) compared to the G3 group (DNA/DNA/DNA), that of differences (*P* < .01) of the G2 group compared to the G4 group (BCG/DNA/DNA), or the G5 group (DNA/DNA/BCG). (b) Mouse protection studies using the prime-boost method. Groups of mice vaccinated with HVJ-E DNA and/or BCG were challenged by intravenous injection with *M. tuberculosis* H37Rv. Five weeks after challenge, protection was measured by enumerating the bacterial loads (CFU) in the liver. Results are expressed as the mean log_10_ ± S.D. of CFU. The statistical significance of differences between individual groups in the CFU number was determined by Dunnett test (*n* = 5), *P < .01; the statistical significance of differences (*P* < .01) of the G1 (naive) group compared to the G4 group (BCG/DNA/DNA), or the G5 group (DNA/DNA/BCG). The statistical significance of differences (*P* < .05) of the G2 group (BCG-alone group) compared to the G4 group (BCG/DNA/DNA). The statistical significance of differences(*P* < .01) of the G2 group compared to the G5 group (DNA/DNA/BCG). The statistical significance of differences (*P* < .05) of the G3 group (DNA/DNA/DNA) compared to G4 (BCG/DNA/DNA). That of differences (*P* < .01) of the G3 group compared to the G5 group. (c) Mouse protection studies using the prime-boost method. Groups of mice vaccinated with HVJ-E DNA and/or BCG were challenged by intravenous injection with *M. tuberculosis* H37Rv. Five weeks after challenge, protection was measured by enumerating the bacterial loads (CFU) in the spleen. Results are expressed as the mean log_10_ ± S.D. of CFU. The statistical significance of differences between individual groups in the number of CFU was determined by Dunnett test (*n* = 5); **P < .05; the statistical significance of differences (*P* < .05) of the G1 (naive) group compared to the G4 group (BCG/DNA/DNA).

At 5 and 10 weeks after intravenous challenge of *M. tuberculosis* H37Rv, the number of CFUs in the lungs, spleen, and liver were determined.  Figure 3(a) shows the result of bacterial loads 5 weeks after challenge.

Vaccination by BCG prime + IgHSP65 + mIL-12/HVJ-E boost showed significant protective effects on the bacterial loads in the lungs as compared to BCG alone (*P* < .01). The prime-boost method using DNA and BCG vaccines showed extremely strong protective efficacy (>10,000-fold versus BCG alone) regardless of the order of administration (Figure 3(a)). Vaccination with BCG vaccine alone decreased TB CFUs in the lungs by 1 log unit as compared to nonvaccinated mice.

Vaccination with IgHSP65 + mIL-12/HVJ-E and BCG by the prime-boost method also showed significant protective efficacy on the bacterial loads in the liver as compared to BCG (>100-fold, *P* < .05; Figure 3(b)). The combination of 2 vaccines and administration by the prime-boost method also exerted a significant protective effect on the bacterial load in the spleen as compared to naive control group (10-fold higher, *P* < .05; Figure 3(c)).

**Figure 4:**
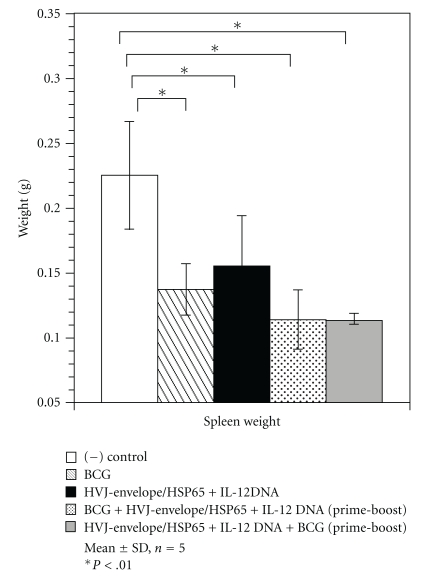
Tissue weight in mouse protection studies using the prime-boost method. Groups of mice vaccinated with HVJ-E DNA and/or BCG were challenged by intravenous injection with *M. tuberculosis* H37Rv. Five weeks after challenge, spleen weight was measured. Results are expressed as the mean ± S.D. in grams (g). The statistical significance of differences between individual groups in the weight was determined by Dunnett test (*n* = 5), *P ≤ .01; the statistical significance of differences (*P* < .01) of the G1 (naive) group compared to the G2 group (BCG-alone group), G3 group (DNA/DNA/DNA), G4 group (BCG/DNA/DNA), or G5 group (DNA/DNA/BCG).

**Figure 5:**
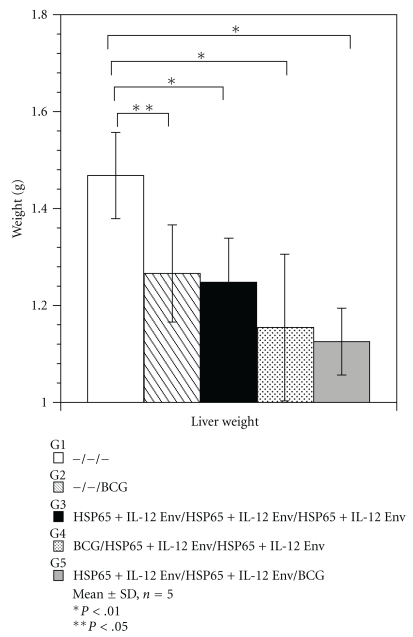
Tissue weight in mouse protection studies using the prime-boost method. Groups of mice vaccinated with HVJ-E DNA and/or BCG were challenged by intravenous injection with *M. tuberculosis* H37Rv. Five weeks after challenge, liver weight was measured. Results are expressed as the mean ± S.D. in grams (g). The statistical significance of differences between individual groups in the weight was determined by Dunnett test (*n* = 5), *P < .01; the statistical significance of differences (*P* ≤ .01) of the G1 (naive) group compared to the G3 group (DNA/DNA/DNA), G4 group (BCG/DNA/DNA), or G5 group (DNA/DNA/BCG). **P < .05; that of differences (*P* < .05) of the G1 group compared to the G2 group (BCG alone group).

Body weight of vaccinated mice was similar in all vaccinated groups. Tissue weights of spleens and livers in the prime-boost groups were lower than those of naive group (Figures 4 and 5).

We also confirmed the greater enhancement of protective effects in the BCG-DNA vaccine combination groups than those in the naive control group or BCG-alone group 10 weeks after challenge (data not shown). These results indicate that treatment using 2 vaccines by the prime-boost method was more effective than BCG alone.

#### 3.1.2. Histological Analysis

**Figure 6:**
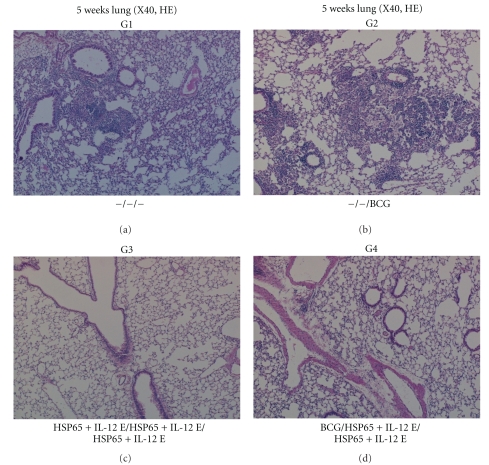
Histopathological analysis of vaccinated mice 5 weeks after *M. tuberculosis* challenge. Representative photomicrographs of lung tissue sections harvested from the G1 naive control group, G2 (BCG alone) group, G3 group (DNA/DNA/DNA), and G4 group (BCG/DNA/DNA) are shown (5 weeks after *M. tuberculosis* challenge, hematoxylin and eosin staining, × 4 objective). There was much infiltration of mononuclear cells and extensive parenchymal destruction by large, poorly demarcated granuloma in the lungs from the G1 (naive control) group and G2 (BCG alone) group. In the G3 (DNA/DNA/DNA) group and G4 (BCG/DNA/DNA) group, there was less inflammation, and only a few granulomas were observed.

**Figure 7:**
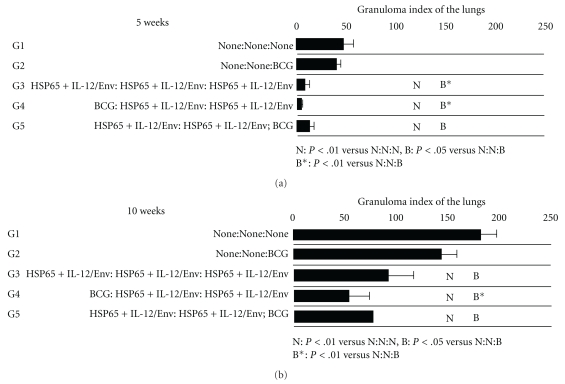
Granuloma index of the G1, G2, G3, G4, and G5 (DNA/DNA/BCG) groups in the lungs 5 weeks and 10 weeks after *M. tuberculosis* challenge. Results are expressed as the mean ± S.D. of triplicates of 5 mice per group. The statistical significance of differences between the groups was determined by Dunnett test, *P* < .01 as compared with the naive (N) group or the BCG alone (B) group. *P* < .05 as compared with the BCG alone (B*) group. The statistical significance of differences (*P* < .05) of granuloma index of 5 weeks G3 group compared to the G4 group.

In addition to the reduction of bacterial loads, the efficacies of each vaccine were assessed by histological analysis. The number and size of granulomatous lesions in the lungs were significantly lower and smaller, respectively, in the mice vaccinated by the BCG prime-DNA boost group than in the naive control mice and BCG control mice groups (Figure 6). Quantitative evaluation of the granulomatous lesions clearly showed that the BCG prime with IgHSP65 + mIL-12/HVJ-E boost significantly reduced the granuloma index in the lungs as compared to naive and BCG groups (*P* < .05; Figure 7). Thus, vaccination by the prime-boost method has the capability to reduce pulmonary lesions caused by *M. tuberculosis* infection.

#### 3.1.3. Immunological Analysis

**Figure 8:**
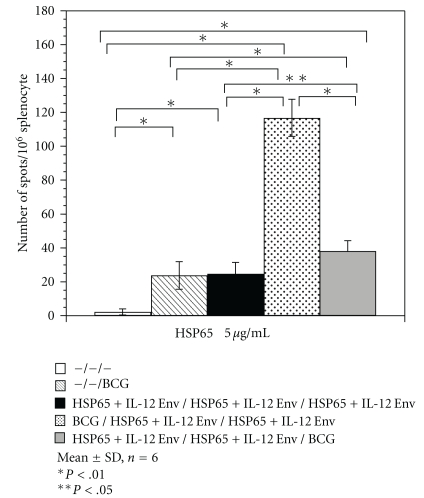
ELISPOT assay for IFN-*γ* antigen-specific responses in the spleens of vaccinated mice following stimulation with rHSP65 protein. Spleen cell cultures were stimulated with rHSP65 protein for 20 h. The numbers of IFN-*γ*-secreting cells specific for rHSP65 protein per million cells were determined individually by ELISPOT assay. Results are expressed as the mean ± S.D. of 6 wells of 3 mice per group. The statistical significance of differences between individual groups in the number of IFN-*γ*-secreting cells was determined by Dunnett test. The statistical significance of differences (*P* < .01) of the G1 (naive) group compared to the G2 (BCG alone group), G3 (DNA/DNA/DNA), G4 (BCG/DNA/DNA), or G5 (DNA/DNA/BCG). The statistical significance of the G2 group difference (*P* < .01) compared to the G4 or the G5. The statistical significance of the G3 group differences (*P* < .01) compared to the G4. *P* < .01; the G4 group compared to the G5. The statistical significance of the G3 group differences (*P* < .05) compared to the G5.

Furthermore, BCG prime with IgHSP65 + mIL-12/HVJ-E boost augmented the proliferation and IFN-*γ* production of HSP65 antigen-specific T cells in the K-S ELISPOT Assay. The efficacy of BCG prime with IgHSP65 + mIL-12/HVJ-E boost was higher compared with BCG Tokyo alone or IgHSP65 + mIL-12/HVJ-E prime with BCG boost (Figure 8).

These data indicate that the protective efficacies of BCG prime with IgHSP65 + mIL-12/HVJ-E boost are strongly associated with the number and activity of IFN-*γ*-secreting and HSP65-specific T cells. Taken together, combinational vaccination with BCG and IgHSP65 + mIL-12/HVJ-E by the prime-boost method is capable of augmenting T-cell activation. In addition, increase of IFN-*γ*-secreting cells is involved in the reduction of bacterial burden and lesions in the lungs. The efficacies of the prime-boost method are greater than those achieved by vaccination with BCG alone.

### 3.2. Discussion

In this study, we evaluated the protective efficacy of IgHSP65 + mIL-12/HVJ-E vaccine, using the prime-boost method. One of the significant findings was that the combination of IgHSP65 + mIL-12/HVJ-E and BCG led to a remarkably high degree of protection against intravenous challenge infection with virulent *M. tuberculosis*; bacterial numbers exponentially declined in 3 organs, especially in the lungs (10,000-fold lower than that of mice vaccinated with BCG alone; Figure 3(a)). 

The pathological parameters of protection included reductions in the mean lung granulomatous lesion score in our study. The protective efficacies of BCG with IgHSP65 + mIL-12/HVJ-E administered by the prime-boost method were indicated on the basis of histopathological methods as well as bacterial loads. Histopathological analysis showed that mice vaccinated with BCG prime with IgHSP65 + mIL-12/HVJ-E boost had fewer and smaller lesions in the lungs and significantly less lung granuloma than naive mice and mice treated with BCG alone. These results suggest that severe toxicities (Koch phenomenon) were suppressed by the combination of two kinds of vaccines.

The data in the present study also show that the protective efficacy of BCG prime with IgHSP65 + mIL-12/HVJ-E boost is strongly associated with the emergence of IFN-*γ*-secreting T cells upon stimulation with HSP65. In the previous study, we demonstrated that *in vivo* function of CD8-positive T cells as well as CD4-positive T cells is involved in prophylactic efficacy of the IgHSP65 + mIL-12/HVJ-E in mice [22]. 

In this study, we used the murine model of TB, which may not reflect the pathologic status of human TB. As to the difference of the infection route, our previous results in a guinea pig model used in a collaborative study with Dr. D. McMurray (Texas A&M University) showed that vaccination with HSP65 + guinea pig IL-12/HVJ resulted in better protection against pulmonary pathology caused by aerosol challenge with *M. tuberculosis* than BCG vaccination (data not shown). 

In addition, we have recently confirmed that the prime-boost method was also effective in a cynomolgus monkey model [20–22]. We evaluated our HSP65 + human IL-12/HVJ (HSP65 + hIL-12/HVJ) in the monkey model infected by an intratracheal instillation (aerogenic route), which is currently the best animal model of human TB. Vaccination with HSP65 + hIL-12/HVJ resulted in better protective efficacy than that with BCG alone on the basis of the erythrocyte sedimentation rate test, chest X-ray findings, and immune responses. In addition, vaccination with HSP65 + hIL-12/HVJ resulted in increased survival for over a year. This was the first report of successful DNA vaccination against M. tuberculosis in a monkey model [21].

Most importantly, protective efficacy was augmented when BCG and HSP65 + hIL-12/HVJ were administered by the prime-boost method. Survival rates of BCG alone, saline control, HSP65 + hIL-12/HVJ-prime with BCG-boost, and BCG-prime with HSP65 + hIL-12/HVJ-boost groups were 33%(2/6), 50%(3/6), 50%(2/4), and 100%(4/4) at 12 months after the infection (aerogenic route), respectively [21]. We also evaluated immune responses in the monkey model of TB. Antigen-specific IFN-*γ*-production and proliferation of peripheral blood lymphocyte (PBL) were enhanced by the vaccination using the prime-boost method. 

We also demonstrated efficacies in the monkey model when the boost was performed after a long-term period (4 months) from the prime. The prolongation of the survival was observed in the BCG-prime and HSP65 + IL-12/HVJ-booster group [27]. Improvement of ESR, increase of the body weight and augmentation of IFN-*γ* production, and proliferation of PBL were also observed in the BCG-prime and HSP65 + IL-12/HVJ-booster group.

Taken together, these results clearly demonstrated that BCG-prime with HSP65 + hIL-12/HVJ-boost could provide extremely strong protective efficacy against M. tuberculosis in a cynomolgus monkey model (intratracheal infection route), which is currently the best animal model of human TB [21].

The prime-boost method was reported in a study of the MVA85A vaccine, which is a modified vaccinia virus Ankara (MVA) strain expressing antigen 85A. In phase I studies in humans, this vaccine has induced high immune responses in previously BCG-vaccinated individuals [28]. Boosting BCG vaccination with MVA85A downregulates the immunoregulatory cytokine TGF-*β*1 [29]. Aeras-402 DNA (DNA that expressed 85A, 85B, and TB10.4) vaccine using adenovirus vector is intended for use as a boosting vaccine in BCG-primed individuals [30]. Several vaccines have been used with a prime-boost strategy to complement immune responses [31].

DNA vaccines are a relatively new approach to induce immunities for the protection of infectious diseases [14, 19, 22, 32–34]. Prophylactic and therapeutic DNA vaccines were established by using several kinds of vectors such as HVJ-liposome, HVJ-E, adenovirus vector, adenoassociated virus vector, and lentivirus vector [19–22, 35, 36]. In order to explore the preclinical use of a tuberculosis DNA vaccine combination of *IL-12* DNA with *hsp65* DNA, we chose the HVJ-based delivery system (HVJ-liposome and HVJ-E). These systems have high transfection efficiency and are available for repeated *in vivo* gene transfection without reduction of gene transfer efficiency or apparent toxicity. These characters of HVJ-liposomes support the feasibility of its clinical application not only for cancer gene therapy but also for DNA vaccinations. In a recent study, highly efficient gene expression in muscle cells was observed for several weeks when pcDNA3 plasmid containing the human tumor antigen genes, *MAGE-1* and *MAGE-3*, were encapsulated in HVJ-liposomes and injected intramuscularly in mice [37]. Effective induction of CD4^+^ T-cell responses by a hepatitis B core particle-based HIV vaccine was achieved by subcutaneous administration of HVJ-liposomes in mice [38]. HVJ-liposomes were also very effective as a mucosal vaccine against HIV infection [39]. Thus, it is likely that HVJ proteins may be responsible for the induction of a robust immune response. No side effects were observed when repetitive injections of HVJ-liposomes were performed in mice, rats, or monkeys. We have previously developed an HVJ-E using inactivated Sendai virus, as a nonviral vector for drug delivery [40–42]. It can be used for efficient delivery of DNAs, siRNAs, proteins, and anticancer drugs into cells both *in vitro* and *in vivo* [40, 43, 44]. Therefore, HVJ-E was used as an efficient and safe vector for DNA vaccine against TB in the present study. 

Mycobacterial heat shock protein 65 (HSP65) is a potential target for protective immunity and has been studied extensively [19]. Several studies have reported that *hsp65* DNA vaccines can strongly induce protective immune responses in mice against virulent *M. tuberculosis* infections [20–22]. Protection is attributed to the establishment of a cellular immune response dominated by HSP65-specific T cells which produce IFN-*γ* and are cytotoxic towards infected cells. Furthermore, Lowrie and colleagues have reported that this vaccine reduces bacterial loads in mice infected with *M. tuberculosis* when given therapeutically after infection [32]. 

One of the major roles of IL-12 is the induction of IFN-*γ*-mediated immune responses to microbial pathogens. Cooper and colleagues have demonstrated the importance of IL-12 in generation of the protective response to tuberculosis [45]. Coadministration of the *IL-12* gene, which induces an IFN-*γ*-mediated immune response to microbial pathogens, with various tuberculosis DNA vaccines including *hsp65* DNA [46], and 35 K MW DNA [47], may boost the efficacy of these DNA vaccines to the levels achieved with BCG in the mouse model, although an inhibitory effect rather than a synergistic effect on immunotherapy was observed in mice coadministered *hsp65* DNA vaccine plus the *IL-12* gene [32]. 

In conclusion, we have shown efficacy of a novel HVJ-E DNA vaccine encapsulating HSP65 DNA with IL-12 DNA in the mouse model of TB. These results suggest that HSP65 + IL-12/HVJ could be a promising candidate for a new tuberculosis vaccine superior to BCG. To this aim, protective efficacy and immune responses were further studied in nonhuman primates before proceeding to human clinical trials.

In Japan and other countries, BCG is inoculated into human infants up to 6 months after birth. Therefore, BCG prime in infants and HSP65 + hIL-12/HVJ boost in adults (including junior high school students, high school students, and the elderly) may be required for significant improvement of clinical protective efficacy against TB. Thus, our results with the HSP65 + hIL-12/HVJ vaccine in a murine prophylactic model and cynomolgus monkey prophylactic model provide a significant rationale for moving this vaccine into clinical trials. Indeed, multiple animal models are available to accumulate essential data on the HVJ-E DNA vaccine in anticipation of a phase I clinical trial.

## 4. Conclusions

Vaccination by BCG prime with a novel vaccine (IgHSP65 + mIL-12/HVJ-E) boost resulted in significant protective efficacy (10,000-fold greater than BCG alone) against TB infection in the lungs of mice. In addition to bacterial loads, significant protective immunity was demonstrated by histopathological analysis of the lungs. This vaccine showed extremely significant protection against TB, suggesting that further development for eventual testing in clinical trials may be warranted.

## Figures and Tables

**Table 1:** BCG-HVJ-E/HSP65 DNA + IL-12 DNA Prime/Boost Experiment. Groups of mice were vaccinated 2 or 3 times with IgHSP65 + mIL-12/HVJ-E vaccine and/or BCG by using the prime-boost method. IgHSP65 + mIL-12/HVJ-E vaccine was injected intramuscularly, and BCG was injected subcutaneously. 4 weeks after the last immunization, *M. tuberculosis *H37Rv was challenged intravenously. 5 weeks and 10 weeks after TB challenge, protection was measured by enumerating bacterial loads (CFU) in the lungs, liver, and spleen from vaccinated mice. One week before the TB challenge, the immune responses of cytotoxic T cells, proliferation of T cells, and cytokines (IFN- *γ*, IL-2, IL-6) production were assayed.

Group	First immunization	Second immunization	Third immunization
1	—	—	—
2	—	—	BCG
3	HSP65 + IL-12/HVJ-E	HSP65 + IL-12/HVJ-E	HSP65 + IL-12/HVJ-E
4	BCG	HSP65 + IL-12/HVJ-E	HSP65 + IL-12/HVJ-E
5	HSP65 + IL-12/HVJ-E	HSP65 + IL-12/HVJ-E	BCG

13 mice per group.

3 mice for the *in vitro* assay prior to challenge (IFN-*γ* ELISPOT, etc.).

10 mice for the protection study (half of the mice were used for necropsy at 5 weeks after challenge and half at 10 weeks).
